# Millisecond timescale reactions observed via X-ray spectroscopy in a 3D microfabricated fused silica mixer. Corrigendum

**DOI:** 10.1107/S1600577522002806

**Published:** 2022-04-05

**Authors:** Diego A. Huyke, Ashwin Ramachandran, Oscar Ramirez-Neri, Jose A. Guerrero-Cruz, Leland B. Gee, Augustin Braun, Dimosthenis Sokaras, Brenda Garcia-Estrada, Edward I. Solomon, Britt Hedman, Mario U. Delgado-Jaime, Daniel P. DePonte, Thomas Kroll, Juan G. Santiago

**Affiliations:** a Stanford University, Stanford, CA 94305, USA; b University of Guadalajara, 44430 Guadalajara, Mexico; c Stanford Synchrotron Radiation Lightsource, SLAC National Accelerator Laboratory, Stanford University, Menlo Park, CA 94025, USA; d Linac Coherent Light Source, SLAC National Accelerator Laboratory, Stanford University, Menlo Park, CA 94025, USA

**Keywords:** microfluidics, mixing, X-ray spectroscopy, kinetics, 3D microfabrication

## Abstract

A figure in the article by Huyke *et al.* [(2021), *J. Synchrotron Rad.* **28**, 1100–1113] is corrected.

The length of the chip was labeled incorrectly in Fig. 9 of the original publication ▸. Specifically, the 12.8 mm dimension applies for the entire length of the chip. For convenience, we also show here the locations of the three inlet ports (these were shown correctly in the original CAD-file of the supporting information). Note that this correction does not influence the discussions or conclusions. The correct figure is shown below. ▸

## Figures and Tables

**Figure 9:**
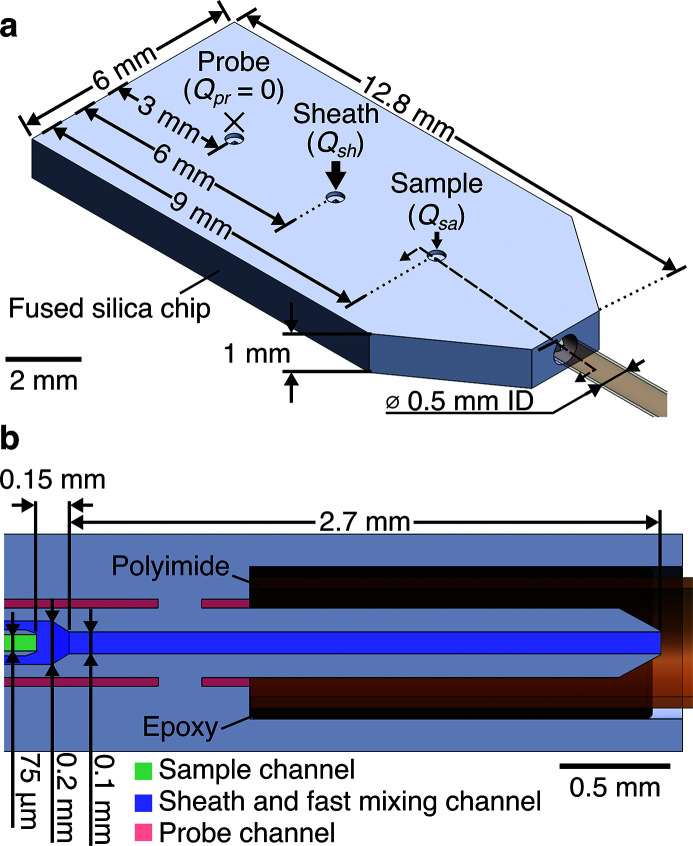
CAD-renderings of microfluidic mixer. (*a*) Isometric view of fused silica chip-polyimide capillary subassembly with key dimensions labeled. The three inlets to the chip are labeled. (*b*) Cross-sectional view at the location of the dashed line in (*a*). The three channels inside of the fused silica chip are highlighted for clarity.

## References

[bb1] Huyke, D. A., Ramachandran, A., Ramirez-Neri, O., Guerrero-Cruz, J. A., Gee, L. B., Braun, A., Sokaras, D., Garcia-Estrada, B., Solomon, E. I., Hedman, B., Delgado-Jaime, M. U., DePonte, D. P., Kroll, T. & Santiago, J. G. (2021). *J. Synchrotron Rad.* **28**, 1100–1113. 10.1107/S1600577521003830 PMC8284405 34212873

